# Correction: Frankenstein, thematic analysis and generative artificial intelligence: Quality appraisal methods and considerations for qualitative research

**DOI:** 10.1371/journal.pone.0337734

**Published:** 2025-11-25

**Authors:** Tanisha Jowsey, Peta Stapleton, Shawna Campbell, Alexandra Davidson, Cher McGillivray, Isabella Maugeri, Megan Lee, Justin Keogh

S1 Table, S2 Table and S1 Fig are incorrect. Please view the correct S1 Table, S2 Table and S1 Fig below.

## Supporting information

S1 Table Reported quotes by human researchers and genAI (Copilot). (PDF)

S2 Table Thematic analysis: Human compared with GenAI Copilot thematic analysis. (PDF)

S1 Fig Human compared with Copilot accuracy of participant/document quotes. (PDF)

## References

[1] Jowsey T, Stapleton P, Campbell S, Davidson A, McGillivray C, Maugeri I, et al. Frankenstein, thematic analysis and generative artificial intelligence: Quality appraisal methods and considerations for qualitative research. PLoS One. 2025;20(9):e0330217. doi: 10.1371/journal.pone.0330217 40911617 PMC12412986

