# Supplementary material for: Frankenstein, thematic analysis and generative artificial intelligence: Quality appraisal methods and considerations for qualitative research
Source: PLoS One. 2025 Sep 5;20(9):e0330217. doi: 10.1371/journal.pone.0330217 (PMC12412986; doi:10.1371/journal.pone.0330217)
Supplement: S2 Table — (DOCX) [file pone.0330217.s002.docx]

Supporting Information File: S2 Fig


S2 Fig. Human compared with Copilot accuracy of participant/document quotes
