# Supplementary material for: Correction: Frankenstein, thematic analysis and generative artificial intelligence: Quality appraisal methods and considerations for qualitative research
Source: PLoS One. 2025 Nov 25;20(11):e0337734. doi: 10.1371/journal.pone.0337734 (PMC12646467; doi:10.1371/journal.pone.0337734)
Supplement: S1 Table — (PDF) [file pone.0337734.s001.pdf]

**Supporting Information File: S1 Table.**

**S1 Table. Reported quotes by human researchers and genAI (Copilot)**

| Study | Absolute score (n) | | Relative score (%) | |
|---|---|---|---|---|
| | Published results | Copilot results | Published results | Copilot results |
| **Quotes correct** | | | | |
| Aurora | 13 | 2 | 41.9% | 20.0% |
| Barlow | 22 | 6 | 100.0% | 60.0% |
| Dunn | 57 | 0 | 95.0% | 0.0% |
| Hervey | 2 | 0 | 100.0% | 0.0% |
| Merkel | 7 | 14 | 58.3% | 100.0% |
| Overall Mean ± SD | 20.2 ± 21.9 | 4.4 ± 5.9 | 79.1 ± 27.1% | 36.0 ± 43.4% |
| | | | | |
| **Quotes modified** | | | | |
| Aurora | 8 | 0 | 25.8% | 0.0% |
| Barlow | 0 | 2 | 0.0% | 20.0% |
| Dunn | 3 | 1 | 5.0% | 12.5% |
| Hervey | 0 | 0 | 0.0% | 0.0% |
| Merkel | 0 | 0 | 0.0% | 0.0% |
| Overall Mean ± SD | 2.2 ± 3.5.5 | 0.6 ± 0.9 | 6.2 ±11.2% | 6.5 ± 9.3% |
| | 3.5 | | | |
| **Quotes fabricated** | | | | |

| | | | | |
|---|---|---|---|---|
| Aurora | 10 | 8 | 32.3% | 80.0% |
| Barlow | 0 | 2 | 0.0% | 20.0% |
| Dunn | 0 | 7 | 0.0% | 87.5% |
| Hervey | 0 | 16 | 0.0% | 100.0% |
| Merkel | 5 | 0 | 41.7%* | 0.0% |
| Overall Mean ± SD | 3 ± 4.5 | 6.6 ± 6.2 | 14.8 ±20.5% | 57.5 ±44.5% |

*Merkel et al. made 15 interview transcripts available for analysis. We could not identify many of their reported quotes from the 15 interview transcripts and it is possible that the quotes come from the remaining transcripts that were not provided. This percentage should be considered with caution.
