# Supplementary material for: Correction: Frankenstein, thematic analysis and generative artificial intelligence: Quality appraisal methods and considerations for qualitative research
Source: PLoS One. 2025 Nov 25;20(11):e0337734. doi: 10.1371/journal.pone.0337734 (PMC12646467; doi:10.1371/journal.pone.0337734)
Supplement: S2 Table — (PDF) [file pone.0337734.s002.pdf]

Supporting Information File: S2 Table.

S2 Table. Thematic analysis: Human compared with GenAI Copilot thematic analysis

	**Human researcher analysis**	**GenAI – first Copilot output**<br>(searches conducted in September 2024)
**Study Arora et al.,**		
Number of participants identified	N = 31	Does not say
Identified themes (number of participants)<br><br>**Overlap ~ 60%**	Thematic analysis<br>Five themes:<br>• Implementation of peer education program<br>• Connectivity between Health Programme-Rashtriya Kishor Swasthya Karyakram (RKSK) health workers and Peer Educators (PEs) and between PEs and adolescents during COVID-19 lockdown.<br>• Effect of COVID-19 on adolescent health services.<br>• Repurposing of RKSK health workers and PEs to support COVID-19 response: During and post lockdown<br>• Adolescents' health and development issues during COVID-19	Five themes:<br>• Implementation and challenges of the RKSK program<br>• Impact of COVID-19 on program activities.<br>• Health issues faced by adolescents.<br>• Role of Peer Educators (PEs).<br>• Differences in health issues between tribal and non-tribal adolescents.

Supporting evidence: data used to support interpretation	Familiarization – two authors report they read transcripts several times.<br><br>Coding – data coded and organized using thematic analysis independently and then discrepancies discussed until consensus reached with a supervising researcher.<br><br>Themes – inductive and deductive steps to derive themes and sub-themes.	Characteristics and reflexivity of the researchers is unchanged.<br><br>Analytical process is opaque.
Additional techniques to enhance trustworthiness (SRQR COREQ Standard)	The stages of thematic analysis are not detailed in the manuscript and no other technique used to enhance trustworthiness.	Analytical process is opaque.<br><br>Clear audit trail not provided.
**Study Barlow et al.,**	**Human researcher analysis**	**GenAI – first Copilot output**
Number of participants identified	N = 47 documents	Does not say
Supporting evidence: data used to support interpretation	Written policy notifications outlining a policy (n = 11)<br><br>TBT meeting minutes (n = 24)<br><br>Written comments on WTO notifications submitted ahead of TBT meetings (n = 12).<br><br>Content: 8 nutrition policies across 7 countries: Thailand, Chile, Indonesia, Peru, Ecuador, Bolivia and Uruguay.	Quotes not supported with meeting minute identifiers.

Identified themes (number of participants)<br><br>Overlap **50%**	Thematic analysis<br>Four Discursive Themes:<br><br>• Neoliberal narratives: individualizing the causes of dietary diseases<br>• Contesting and affirming congruence with neoliberal economic theory, values and beliefs<br>• Re-defining policy goals to ensure congruence with neoliberal economic theory, values, and beliefs<br>• Setting policy norms by appealing to high-income members' authority	Five themes:<br><br>• Effectiveness of Labelling Systems<br>• Scientific and Technical Justifications<br>• Trade and Economic Implications<br>• Consumer Perception and Behavior<br>• Regulatory Compliance and Challenges<br><br>These themes are micro level (what was said) whereas the human researchers' themes are macro (what it's about and how it relates to socio-cultural contexts).<br><br>Very small overlap in thematic material.
Evidence of analytical process: Context described and taken account of in interpretation.	Critical Discourse Analysis: an approach that "explores often opaque relationships between discursive practices, events and texts and wider social and cultural structures, relations and processes … and [how] these arise out of and are ideologically shaped by relations of power and struggles over power."<br><br>Exploration of these relationships occurred by combining a thematic analysis of the content of Technical Barriers to Trade, discussions with consideration of the power sources and relations in World Trade Organization members' arguments.	Characteristics and reflexivity of the researchers is unchanged.<br><br>Analytical process is opaque.

Additional techniques to enhance trustworthiness (SRQR COREQ Standard)	Reflexivity statement: one sentence.<br><br>Authors also describe the theoretical and empirical background of their study, their analytical focus and how they integrated it into the analysis.<br><br>Two coders.	Analytical process is opaque.<br><br>Clear audit trail not provided.
**Study Dunn et al., 2021**	**Dunn human researcher analysis**	**Gen AI – first Copilot output** [05.09.2024 at 3.33pm Standard Eastern Time]
Number of participants identified	N=33<br>Parents (n=12)<br>Practitioner (n=21)	Does not say.

Identified themes (number of participants)<br><br>**Overlap ~?**	Thematic analysis<br>10 Themes<br>18 Subthemes<br><br>• Impact of mental health difficulties<br>○ Emotional Intensity<br>Coping strategy: Façade<br>• Impact of trauma<br>○ Lack of parenting model<br>○ experience<br>Legacy of abuse<br>Negative view of self as parent<br>• Unsupported parenting<br>○ Social and family network<br>• Professional support<br>• Self in relation to child<br>• Lack of insight<br><br>Note, several themes (4 Themes and 6 Subthemes) concerned both parents and practitioners. One Subtheme (3.a) concerned solely parents. Two themes (5 & 6) and one Subtheme (1.c) concerned solely practitioners.	Eight themes:<br><br>Concerning practitioners:<br>Zero themes<br><br>• Challenges of Parenting with Mental Health Issues<br>• Impact of Mental Health on Family Dynamics<br>• Struggles with Support Services<br>• Stigmatization and Biases<br>• Importance of Empathy and Support<br>• Impact of Past Traumas<br>• Need for Early Intervention<br>• Complexities of Domestic Situations<br><br>Note, themes only concerned parents.

Evidence of analytical process: Context described and taken account of in interpretation.	Data were deidentified.<br><br>Framework analysis undertaken.<br><br>Familiarization – two authors report they read transcripts and coded independently.<br><br>Coding – data coded and organized using thematic analysis independently and then discrepancies discussed until consensus reached with a supervising researcher.<br><br>Reflexivity statement:<br>Three sentences provided.<br><br>Parents were invited to meet with the research team to discuss the emergent themes. Four participants met<br>as a group with the lead researcher and provided feedback on the themes<br>identified in the data, which was used to refine and name the themes.	Characteristics and reflexivity of the researchers is unchanged.<br><br>Analytical process is opaque.
Additional techniques to enhance trustworthiness (SRQR COREQ Standard)	The lead researcher and a second researcher separately reviewed the data at each stage of the process and these perspectives were integrated iteratively into the thematic framework and the index. Additional oversight and comments were provided by the lead researcher's supervisory team.	Analytical process is opaque.<br><br>Clear audit trail not provided
**Study Hervey et al., 2021**	**Human researcher analysis**	**GenAI – first Copilot output**
Number of participants identified	N=48	N=15

Identified themes (number of participants)	Discursive thematic analysis<br>4 themes:<br>• Health Brexternalities through time<br>• Spatially differentiated health Brexternalities<br>• EU population-wide health Brexternalities<br>• Global health Brexternalities<br><br>Hervey et al. provide analysis integrated with socio-historical contextual information, legal documents, case law, and white and grey literature. They demonstrate sophisticated understanding of the subject matter. They make few references to participants, only two direct participant quotes in the manuscript (one quote is two words long and the other is three words long).	Eight themes:<br><br>• Impact of Brexit on Healthcare Workforce (N=15)<br>• Reciprocal Healthcare and Patient Safety<br>• Supply Chain and Medical Products<br>• Public Health Law and Standards<br>• Governance and Stakeholder Engagement<br>• Economic and Socio-Economic Effects<br>• Professional Qualifications and Training<br>• Political and Legal Complexities<br><br>Highlighted themes concern law, policy, socio-political context, history, workforce issues and medicine supply chain issues – overlapping with the authors themes but not in terms of time/space/population health.
Evidence of analytical process: Context described and taken account of in interpretation.	• None provided	Characteristics and reflexivity of the researchers is unchanged.<br><br>Analytical process is opaque.

Additional Techniques to enhance trustworthiness (SRQR COREQ Standard)	• None	Analytical process is opaque.<br>Clear audit trail not provided
**Study Merkel et al., 2019**	**Human researcher analysis**	**GenAI – first Copilot output**
Number of participants identified	N = 54<br>54 interviews within 11 case studies: Germany (6), UK (2), Finland (3)	N=15*<br>Output does not report N.<br><br>*human researchers made 15 interview transcripts available for analysis.
Identified themes (number of participants)	Content analysis<br>11 Themes<br><br>Challenge:<br>• Lack of skilled personnel<br>• Difficulty to recruit<br>• Ageing workforce<br>• Short term substitutes<br>• Staff turnover<br>• Demanding working conditions<br>Age management measures:<br>• Tackling problems of scarce resources<br>• Decreasing work demands<br>• Enhancing individual resources<br>• Intergenerational learning<br>• Life-course approach	Seven themes:<br>• Training and development<br>• Workforce flexibility<br>• Health and Wellbeing Support<br>• Challenges of Aging Workforce<br>• Gender dynamics<br>• Career development opportunities<br>• Impact of government policies

Evidence of analytical process: Context described and taken account of in interpretation.	• Exploration of perceptions within the sector<br>• Familiarization with the data – reading manuscript multiple times<br>• Categories generated using deductive-inductive approach – generated categories based on previous research and interviews.<br>• Coding -	Characteristics and reflexivity of the researchers is unchanged.<br><br>Analytical process is opaque.
Additional techniques to enhance trustworthiness (SRQR COREQ Standard)	• Researcher triangulation – two researchers conducted interviews and coding of transcripts.	Analytical process is opaque.<br><br>Clear audit trail not provided.
