# Supplementary material for: Correction: Frankenstein, thematic analysis and generative artificial intelligence: Quality appraisal methods and considerations for qualitative research
Source: PLoS One. 2025 Nov 25;20(11):e0337734. doi: 10.1371/journal.pone.0337734 (PMC12646467; doi:10.1371/journal.pone.0337734)
Supplement: S1 Fig — (PDF) [file pone.0337734.s003.pdf]

Supporting Information File: S1 Fig

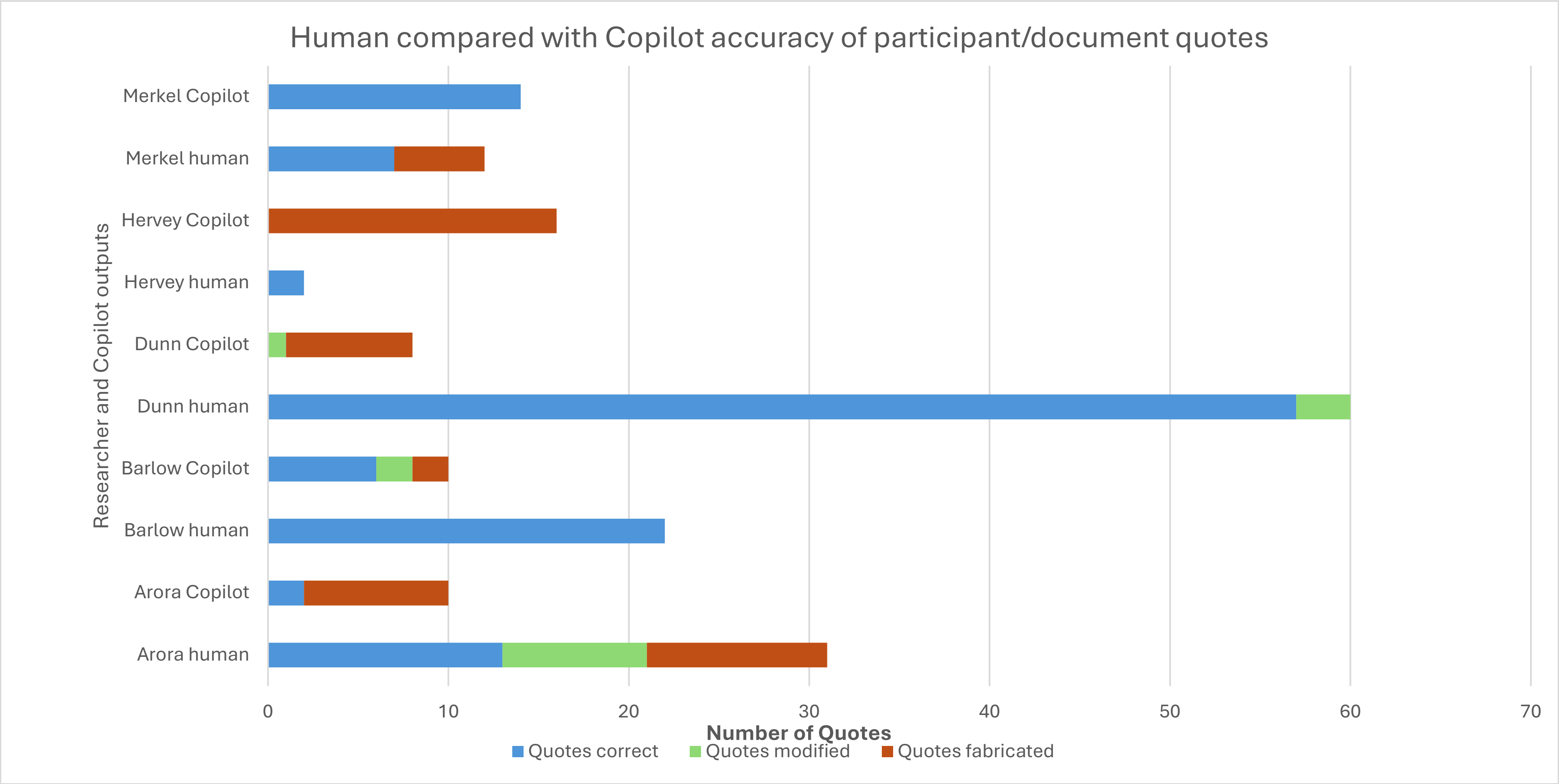

S1 Fig. Human compared with Copilot accuracy of participant/document quotes
